# Thortveitite-type Tm_2_Si_2_O_7_

**DOI:** 10.1107/S1600536814013142

**Published:** 2014-06-11

**Authors:** Volker Kahlenberg, Paul Aichholzer

**Affiliations:** a University of Innsbruck, Institute of Mineralogy & Petrography, Innrain 52, A-6020 Innsbruck, Austria

## Abstract

Single crystals of dithulium disilicate, Tm_2_Si_2_O_7_, were obtained in flux synthesis experiments in the system SiO_2_–Tm_2_O_3_–LiF at ambient pressure. The compound belongs to the group of sorosilicates, *i.e.* it is based on [Si_2_O_7_]-units and crystallizes in the thortveitite (Sc_2_Si_2_O_7_) structure type. The Tm^3+^ cation (site symmetry .2.) occupies a distorted octa­hedral site, with Tm—O bond lengths in the range 2.217 (4)–2.289 (4) Å. Each of the octa­hedra shares three of its edges with adjacent [TmO_6_] groups, resulting in the formation of layers parallel to (001). The individual [SiO_4_] tetra­hedra are more regular, *i.e.* the differences between the bond lengths between Si and the bridging and non-bridging O atoms are not very pronounced. The layers containing the octa­hedra and the sheets containing the [Si_2_O_7_] groups (point group symmetry 2/*m*) form an alternating sequence. Linkage is provided by sharing common oxygen vertices.

## Related literature   

For applications of oxosilicates containing trivalent rare earth elements (*REE*), see: Kitai (2008 ▶); Piccinelli *et al.* (2009 ▶); Qiao *et al.* (2014 ▶); Luo *et al.* (2012 ▶); Streit *et al.* (2013 ▶); Han *et al.* (2006 ▶); Sun *et al.* (2012 ▶). For structures isotypic with that of the title compound, see: Zachariasen (1930 ▶); Smolin *et al.* (1973 ▶); Christensen (1994 ▶); Redhammer & Roth (2003 ▶). For polymorphic forms of Tm_2_Si_2_O_7_ and other structure types adopted by (*REE*)_2_Si_2_O_7_ compounds, see: Bocquillon *et al.* (1977 ▶); Hartenbach *et al.* (2003 ▶); Felsche (1973 ▶); Fleet & Liu (2005 ▶); Shannon & Prewitt (1970 ▶). For discussions of the [Si_2_O_7_]-unit with a linear bridging angle, see: Baur (1980 ▶); Bianchi *et al.* (1988 ▶); Cruickshank *et al.* (1962 ▶); Kimata *et al.* (1998 ▶); Liebau (1961 ▶). For general aspects on the crystal chemistry of silicates, see: Liebau (1985 ▶). For definition of distortion parameters, see: Robinson *et al.* (1971 ▶). For bond-valence analysis, see: Brown (2002 ▶). For definition and calculation of similarity descriptors, see: Tasci *et al.* (2012 ▶); Bergerhoff *et al.* (1999 ▶). For ionic radii, see: Shannon (1976 ▶). For the Inorganic Crystal Structure Database, see: ICSD (2014 ▶).

## Experimental   

### 

#### Crystal data  

Tm_2_Si_2_O_7_
*M* *_r_* = 506.04
Monoclinic, 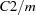

*a* = 6.8205 (14) Å
*b* = 8.9062 (18) Å
*c* = 4.6937 (11) Å
β = 101.78 (2)°
*V* = 279.11 (10) Å^3^
*Z* = 2
Mo *K*α radiation
μ = 31.99 mm^−1^
*T* = 293 K
0.05 × 0.03 × 0.01 mm

#### Data collection  

Agilent Xcalibur (Ruby, Gemini ultra) diffractometer
Absorption correction: multi-scan (*CrysAlis PRO*; Agilent, 2014 ▶) *T*_min_ = 0.231, *T*_max_ = 1
894 measured reflections
340 independent reflections
330 reflections with *I* > 2σ(*I*)
*R*_int_ = 0.020

#### Refinement  

*R*[*F* ^2^ > 2σ(*F* ^2^)] = 0.018
*wR*(*F* ^2^) = 0.045
*S* = 1.15
340 reflections
32 parameters
Δρ_max_ = 1.62 e Å^−3^
Δρ_min_ = −1.42 e Å^−3^

### 

Data collection: *CrysAlis PRO* (Agilent, 2014 ▶); cell refinement: *CrysAlis PRO*; data reduction: *CrysAlis PRO*; program(s) used to solve structure: *SIR2002* (Burla *et al.*, 2003 ▶); program(s) used to refine structure: *SHELXL97* (Sheldrick, 2008 ▶); molecular graphics: *ATOMS for Windows* (Dowty, 2011 ▶); software used to prepare material for publication: *publCIF* (Westrip, 2010 ▶) and *WinGX* (Farrugia, 2012 ▶).

## Supplementary Material

Crystal structure: contains datablock(s) global, I, New_Global_Publ_Block. DOI: 10.1107/S1600536814013142/wm5029sup1.cif

Structure factors: contains datablock(s) I. DOI: 10.1107/S1600536814013142/wm5029Isup2.hkl

CCDC reference: 1006971

Additional supporting information:  crystallographic information; 3D view; checkCIF report

## Figures and Tables

**Fig. 1.**
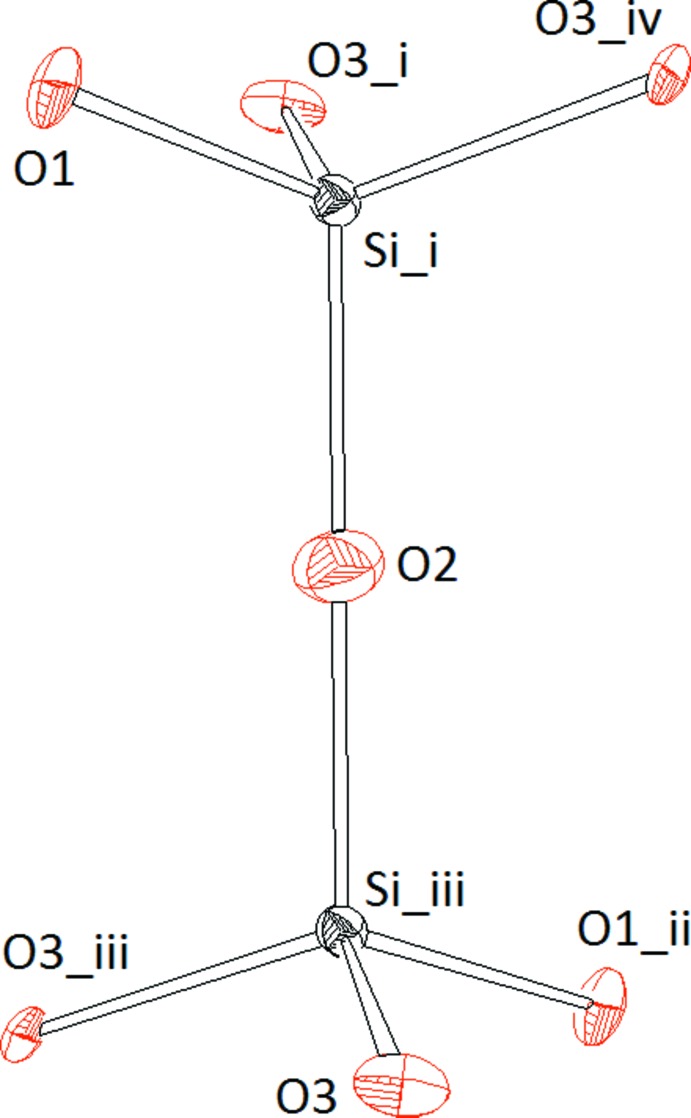
Representation of a single [Si2O7]-unit. Ellipsoids are drawn at the 60% probability level. [Symmetry codes: (i) x, y, -1 + z (ii) -x, y, -z (iii) -x, y, 1 - z (iv) x, -y, -1 + z].

**Fig. 2.**
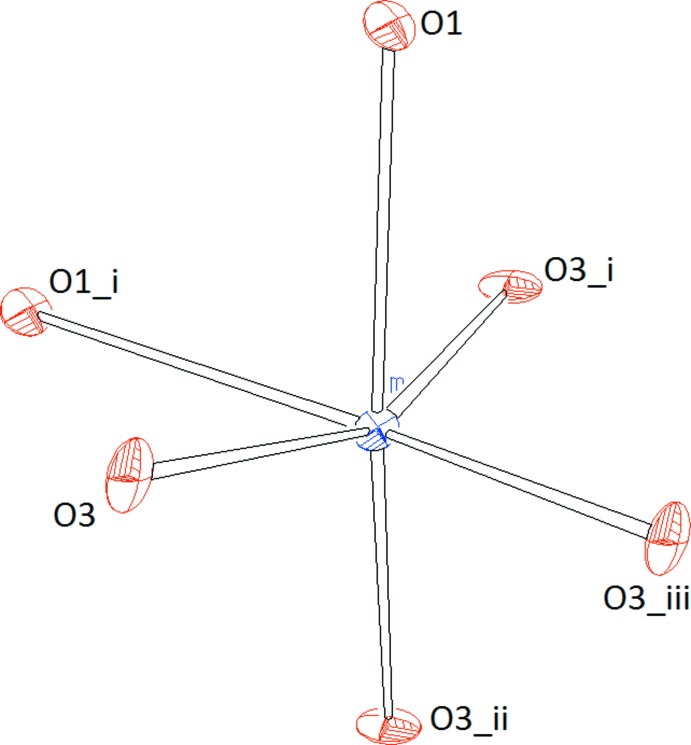
Representation of the coordination around the trivalent Tm ion. Ellipsoids are drawn at the 60% probability level. [Symmetry codes: (i) 1 - x, y, 1 - z (ii) 1/2 + x, 1/2 - y, z (iii) 1/2 - x, 1/2 - y, 1 - z].

**Fig. 3.**
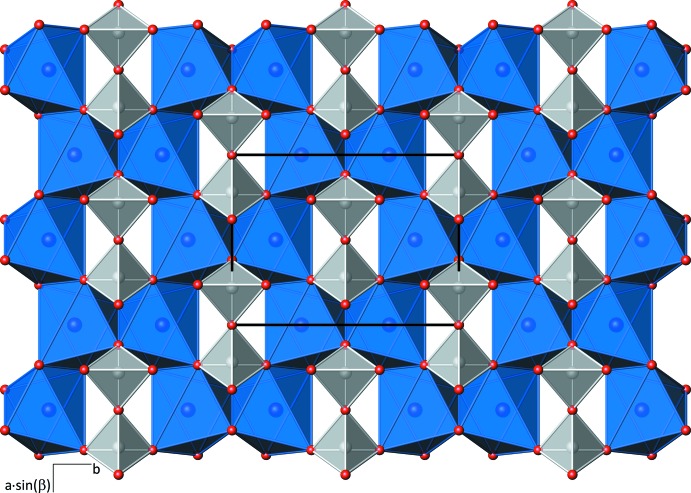
Single layer of edge-sharing octahedra and one of the two adjacent sheets containing [Si2O7]-units in a projection parallel to [001]. Red, grey and blue spheres represent oxygen, silicon and thulium ions.

## supplementary crystallographic information

### S1. Comment

Oxosilicates that contain trivalent rare earth elements have been studied frequently because of their potential usage in the field of luminescense including applications in devices and circuits for electronic, optoelectronic as well as communication industries (Kitai, 2008; Piccinelli *et al.*, 2009; Qiao *et al.*, 2014; Luo *et al.*, 2012; Streit *et al.*, 2013; Han *et al.*, 2006; Sun *et al.*, 2012).

In the course of an ongoing project on the synthesis of alkali-*REE*-silicates (*REE* is a rare earth element), single-crystals of Tm_2_Si_2_O_7_ have been obtained and structurally characterized. Synthetic Tm_2_Si_2_O_7_ is isotypic with thortveitite (Sc_2_Si_2_O_7_), a rare scandium silicate mineral (Zachariasen, 1930; Smolin *et al.*, 1973). The compound is a sorosilicate and contains isolated [Si_2_O_7_]-groups. The bridging oxygen atom of the dimer resides on a centre of symmetry, resulting in a linear Si—O—Si angle. The conformation of the group is staggered with a dihedral angle (or azimuth) of 60° (Fig. 1). In the past, the question whether or not Si—O—Si angles can exhibit a value of 180° has been discussed controversially and, actually, the thortveitite structure-type played an important role in this debate (Liebau, 1961; Cruickshank, *et al.*, 1962). However, a critical analysis of published data performed by Baur (1980) revealed that linear Si—O—Si bridging angles do exist and cannot be attributed to incorrect space group assignments. To date, it is generally accepted that a description of the thortveitite structure-type in the centrosymmetric space group *C*2/*m* (implying a linear Si—O—Si angle) is correct (Bianchi *et al.*, 1988; Kimata *et al.*, 1998). The present structure determination of Tm_2_Si_2_O_7_ also confirms this model. The spread in the Si—O and O—Si—O angles is not very pronounced and the values are in the expected limits for silicates (Liebau, 1985). Numerically, the degree of distortion can be expressed by the quadratic elongation QE and the angle variance AV (Robinson *et al.*, 1971). The values of these distortion parameters for a single [SiO_4_]-tetrahedron are very small: 1.001 (for QE) and 4.95 (for AV), respectively. The Tm^3+^ cations are octahedrally coordinated by O atoms (Fig. 2), with Tm—O bond lengths in the range 2.217 (4) – 2.289 (4) Å and an average of 2.247 Å. The mean value compares well with those observed for the thortveitite representatives of the directly neighbouring *REE* Yb (<Yb—O>=2.240 Å) and Er (<Er—O>=2.253 Å) (Christensen, 1994). The differences can be attributed to the increasing ionic radii of the trivalent cations in the series Yb^3+^ - Tm^3+^ - Er^3+^ (Shannon, 1976). The octahedra show a distortion with moderate QE-values (1.061) and very high values for the angle variance. The high AV value of 219.7 seems to be a characteristic feature of the thortveitite structure-type and has been also observed for other members of this family (Redhammer & Roth, 2003). Each of the octahedra shares three of its edges with adjacent [TmO_6_]-groups resulting in the formation of layers parallel to (001). These pseudo-hexagonal sheets (Fig. 3) are similar to the layers in dioctahedral micas. The above-mentioned pronounced angular distortions can be rationalized by a combination of (i) a shortening of the common edges of adjacent octahedra (in order to reduce the repulsive interactions between adjoining Tm^3+^ cations) and (ii) a widening of the corresponding opposite unshared O—O edges. Successive layers containing octahedra are linked by the [Si_2_O_7_]-groups in such a way that each of both tetrahedra shares two of its corners with two different octahedra from the same and one corner with an octahedron from the other surrounding layer.

Bond valence sum calculations using the parameter sets for the Tm—O and Si—O bonds given by Brown (2002) resulted in the following values (in v.u.) for the cation-anion interactions up to 3.4 Å: Tm: 3.09, Si: 4.01, O1: 2.12, O2: 1.99 and O3: 1.99.

As mentioned above, the present structure is isotypic with that of thortveitite. For the calculation of several quantitative descriptors for the characterization of the degree of similarity between the crystal structures of Tm_2_Si_2_O_7_ and Sc_2_Si_2_O_7_, the program *COMPSTRU* (Tasci *et al.*, 2012) was employed. For the given two structures, the degree of lattice distortion (S), *i.e.* the spontaneous strain obtained from the eigenvalues of the finite Lagrangian strain tensor calculated in a Cartesian reference system, has a value of (S) = 0.0222. After application of an origin shift of p = (0, 0, 1/2) the structure of Tm_2_Si_2_O_7_ was transformed to the most similar configuration of Sc_2_Si_2_O_7._ The calculations revealed the following atomic displacements (in Å) between the corresponding atoms in Sc_2_Si_2_O_7_ (first entry) and Tm_2_Si_2_O_7_ (second entry): Sc—Tm: 0.025; Si—Si: 0.043; O1—O2: 0.000; O2—O1: 0.083; O3—O3: 0.070 *i.e.* the maximum displacement is lower than 0.10 Å. The measure of similarity (Δ) as defined by Bergerhoff *et al.* (1999) has a value of 0.059.

Since the beginning of the 1970ies a large number of different structure types have been described for rare earth element silicates with composition (*REE*)_2_Si_2_O_7_ (Felsche, 1973). To date, at least twelve different forms (*A—I*, *K*, *L* and *X*) have to be distinguished (Fleet & Liu, 2005). Tm_2_Si_2_O_7_, for example, exhibits a high degree of polymorphism where five different modifications can be realised. The synthesis of polycrystalline Tm_2_Si_2_O_7_ adopting the thortveitite- or *C*-type has been described by Bocquillon *et al.* (1977) in the temperature range between 1473 and 1673 K. However, the stability field of *C*-type Tm_2_Si_2_O_7_ extends to higher pressures as well: synthesis runs performed at 65 kbar/1773 K (Shannon & Prewitt, 1970) as well as 10 kbar/973 K and 18 kbar/973 K (Bocquillon *et al.*, 1977) also resulted in the formation of the *C*-phase. Other high-pressure modifications of Tm_2_Si_2_O_7_ crystallize in the *B*–, *D*–, *X*– and *L*-types (Fleet & Liu, 2005; Shannon & Prewitt, 1970). The *B*-type, however, has been also prepared at ambient pressure and 1173 K (Hartenbach *et al.*, 2003). In summary, one can say that more than forty years after the first systematic investigations to chart the *p*,*T*-behaviour of Tm_2_Si_2_O_7_, there are still open questions. The new flux synthesis route using lithium fluoride as a mineralizer offers the possibility to grow large single-crystals suited for *in situ* X-ray diffraction or Raman spectroscopic high-pressure studies in diamond anvil cells.

### S2. Experimental

Starting materials for the flux growth experiments were dried reagent grade Tm_2_O_3_, SiO_2_ and LiF. Due to the pronounced hygroscopicity of the alkali fluoride, sample preparation was performed in a glove bag under nitrogen atmosphere. 0.1 g of the nutrient consisting of a mixture of Tm_2_O_3_:SiO_2_ in the molar ratio 1:4 was homogenized in an agate mortar with 0.1 g LiF. Subsequently, the educts were loaded into a platinum tube with an outer diameter of 3 mm and with 20 mm length. After sealing, the tube and its content were heated in a resistance furnace from 373 K to 1373 K with a rate of 50 K/h and isothermed for 2 h at the target temperature. The sample was cooled down to 1073 K with a rate of 5 K/h and, finally, the temperature was reduced to 373 K with a rate of 100 K/h. Removal of the flux with water left a residue of transparent, colorless, optically biaxial and highly birefringent crystals up to 500 µm in size. One of the optically biaxial crystals showing sharp extinction when observed between crossed polarizers was selected for further structural studies and mounted on the tip of a glass fiber using finger nail hardener as glue.

### S3. Refinement

Similar sets of lattice parameters could be found in the recent WEB-based version of the Inorganic Crystal Structure Database (ICSD, 2014) for the chemically closely related thortveitite-type materials with composition (*REE*)_2_Si_2_O_7_ pointing to an isostructural relationship, which was confirmed by the subsequent structure analysis by direct methods. For structure determination a data set corresponding to a hemisphere of reciprocal space was collected.

### Crystal data

**Table:**

Tm_2_Si_2_O_7_	*F*(000) = 444
*M**_r_* = 506.04	*D*_x_ = 6.021 Mg m^−^^3^
Monoclinic, *C*2/*m*	Mo *K*α radiation, λ = 0.71073 Å
Hall symbol: -C 2y	Cell parameters from 762 reflections
*a* = 6.8205 (14) Å	θ = 3.8–29.3°
*b* = 8.9062 (18) Å	µ = 31.99 mm^−^^1^
*c* = 4.6937 (11) Å	*T* = 293 K
β = 101.78 (2)°	Platy fragment, colourless
*V* = 279.11 (10) Å^3^	0.05 × 0.03 × 0.01 mm
*Z* = 2	

### Data collection

**Table:**

Agilent Xcalibur (Ruby, Gemini ultra) diffractometer	340 independent reflections
Radiation source: Enhance (Mo) X-ray Source	330 reflections with *I* > 2σ(*I*)
Graphite monochromator	*R*_int_ = 0.020
Detector resolution: 10.3575 pixels mm^-1^	θ_max_ = 27.6°, θ_min_ = 3.8°
ω scans	*h* = −8→8
Absorption correction: multi-scan (*CrysAlis PRO*; Agilent, 2014)	*k* = −11→11
*T*_min_ = 0.231, *T*_max_ = 1	*l* = −4→6
894 measured reflections	

### Refinement

**Table:**

Refinement on *F*^2^	Primary atom site location: structure-invariant direct methods
Least-squares matrix: full	Secondary atom site location: difference Fourier map
*R*[*F*^2^ > 2σ(*F*^2^)] = 0.018	*w* = 1/[σ^2^(*F*_o_^2^) + (0.0265*P*)^2^] where *P* = (*F*_o_^2^ + 2*F*_c_^2^)/3
*wR*(*F*^2^) = 0.045	(Δ/σ)_max_ < 0.001
*S* = 1.15	Δρ_max_ = 1.62 e Å^−^^3^
340 reflections	Δρ_min_ = −1.42 e Å^−^^3^
32 parameters	Extinction correction: *SHELXL97* (Sheldrick, 2008), Fc^*^=kFc[1+0.001xFc^2^λ^3^/sin(2θ)]^-1/4^
0 restraints	Extinction coefficient: 0.0072 (6)

### Special details

**Table:**

Geometry. All e.s.d.'s (except the e.s.d. in the dihedral angle between two l.s. planes) are estimated using the full covariance matrix. The cell e.s.d.'s are taken into account individually in the estimation of e.s.d.'s in lengths, angles and torsion angles; correlations between e.s.d.'s in cell parameters are only used when they are defined by crystal symmetry. An approximate (isotropic) treatment of cell e.s.d.'s is used for estimating e.s.d.'s involving l.s. planes.
Refinement. Refinement of *F*^2^ against ALL reflections. The weighted *R*-factor *wR* and goodness of fit *S* are based on *F*^2^, conventional *R*-factors *R* are based on *F*, with *F* set to zero for negative *F*^2^. The threshold expression of *F*^2^ > σ(*F*^2^) is used only for calculating *R*-factors(gt) *etc*. and is not relevant to the choice of reflections for refinement. *R*-factors based on *F*^2^ are statistically about twice as large as those based on *F*, and *R*-factors based on ALL data will be even larger.

### Fractional atomic coordinates and isotropic or equivalent isotropic displacement parameters (Å^2^)

**Table:**

	*x*	*y*	*z*	*U*_iso_*/*U*_eq_	
Tm	0.5	0.19345 (4)	0.5	0.0045 (2)	
Si	0.2186 (3)	0	0.9130 (5)	0.0044 (5)	
O1	0.3804 (9)	0	0.2130 (12)	0.0069 (12)	
O2	0	0	0	0.0129 (19)	
O3	0.2357 (6)	0.1505 (5)	0.7213 (9)	0.0073 (8)	

### Atomic displacement parameters (Å^2^)

**Table:**

	*U*^11^	*U*^22^	*U*^33^	*U*^12^	*U*^13^	*U*^23^
Tm	0.0032 (3)	0.0043 (3)	0.0058 (3)	0	0.00056 (15)	0
Si	0.0049 (11)	0.0043 (11)	0.0042 (11)	0	0.0013 (9)	0
O1	0.006 (3)	0.007 (3)	0.005 (3)	0	−0.004 (2)	0
O2	0.008 (4)	0.019 (5)	0.011 (5)	0	0.001 (4)	0
O3	0.006 (2)	0.007 (2)	0.009 (2)	0.0042 (17)	0.0026 (17)	0.0045 (17)

### Geometric parameters (Å, º)

**Table:**

Tm—O3^i^	2.217 (4)	Si—O3^vi^	1.632 (4)
Tm—O3^ii^	2.217 (4)	Si—O3	1.632 (4)
Tm—O1	2.236 (4)	O1—Si^vii^	1.602 (6)
Tm—O1^iii^	2.236 (4)	O1—Tm^iii^	2.236 (4)
Tm—O3	2.289 (4)	O2—Si^viii^	1.624 (2)
Tm—O3^iv^	2.289 (4)	O2—Si^vii^	1.624 (2)
Si—O1^v^	1.602 (6)	O3—Tm^ii^	2.217 (4)
Si—O2^v^	1.624 (2)		
			
O3^i^—Tm—O3^ii^	102.3 (2)	O3—Tm—O3^iv^	160.7 (2)
O3^i^—Tm—O1	154.9 (2)	O1^v^—Si—O2^v^	106.4 (2)
O3^ii^—Tm—O1	93.47 (17)	O1^v^—Si—O3^vi^	111.8 (2)
O3^i^—Tm—O1^iii^	93.47 (17)	O2^v^—Si—O3^vi^	108.14 (18)
O3^ii^—Tm—O1^iii^	154.9 (2)	O1^v^—Si—O3	111.8 (2)
O1—Tm—O1^iii^	79.2 (2)	O2^v^—Si—O3	108.14 (18)
O3^i^—Tm—O3	117.09 (17)	O3^vi^—Si—O3	110.4 (3)
O3^ii^—Tm—O3	75.78 (17)	Si^vii^—O1—Tm	129.10 (12)
O1—Tm—O3	85.4 (2)	Si^vii^—O1—Tm^iii^	129.10 (12)
O1^iii^—Tm—O3	79.74 (18)	Tm—O1—Tm^iii^	100.8 (2)
O3^i^—Tm—O3^iv^	75.78 (17)	Si^viii^—O2—Si^vii^	180.00 (14)
O3^ii^—Tm—O3^iv^	117.09 (17)	Si—O3—Tm^ii^	130.3 (3)
O1—Tm—O3^iv^	79.74 (18)	Si—O3—Tm	122.6 (2)
O1^iii^—Tm—O3^iv^	85.43 (19)	Tm^ii^—O3—Tm	104.22 (17)

Symmetry codes: (i) *x*+1/2, −*y*+1/2, *z*; (ii) −*x*+1/2, −*y*+1/2, −*z*+1; (iii) −*x*+1, −*y*, −*z*+1; (iv) −*x*+1, *y*, −*z*+1; (v) *x*, *y*, *z*+1; (vi) *x*, −*y*, *z*; (vii) *x*, *y*, *z*−1; (viii) −*x*, −*y*, −*z*+1.
